# Characterizing the penumbras of white matter hyperintensities in patients with cerebral small vessel disease

**DOI:** 10.1007/s11604-023-01419-w

**Published:** 2023-05-09

**Authors:** Xin Wang, Yu Wang, Deyu Gao, Zhichao Zhao, Haiping Wang, Sujie Wang, Shiguang Liu

**Affiliations:** 1 Department of Radiology, Tangshan Gongren Hospital, 27 Wenhua Road, Tangshan City, 063000 Hebei Province China; 2 North China University of Technology, Tangshan City, 063000 Hebei Province China; 3 Department of Neurology, Tangshan Gongren Hospital, 27 Wenhua Road, Tangshan City, 063000 Hebei Province China

**Keywords:** White matter hyperintensity, Normal appearing white matter, Penumbra, Cerebral blood flow, Diffusion kurtosis imaging

## Abstract

**Purpose:**

The white matter hyperintensity penumbra (WMH-P) is the subtly changed normal-appearing white matter (NAWM) that surrounds white matter hyperintensities (WMHs). The goal of this study was to define WMH-P in cerebral small vessel disease (CSVD) by arterial spin labeling (ASL) and diffusion tensor imaging (DTI)/diffusion kurtosis imaging (DKI).

**Materials and methods:**

We prospectively analyzed 42 patients with CSVD. To determine the range of cerebral blood flow (CBF) and DTI/DKI penumbras around white matter hyperintensities, we generated NAWM layer masks from periventricular WMHs (PVWMHs) and deep WMHs (DWMHs). Mean values of CBF, fractional anisotropy, mean diffusivity, axial diffusivity, radial diffusivity, mean kurtosis, axial kurtosis, and radial kurtosis within the WMHs and their corresponding NAWM layer masks were analyzed. Paired sample *t* tests were used for analysis, and differences were considered statistically significant if the associated *p* value was ≤ 0.05.

**Results:**

For DWMHs, the CBF penumbras were 13 mm, and the DTI/DKI penumbras were 8 mm. For PVWMHs, the CBF penumbras were 14 mm, and the DTI/DKI penumbras were 14 mm.

**Conclusions:**

Our findings revealed that DTI/DKI and ASL can show structural and blood flow changes in brain tissue surrounding WMHs. In DWMHs, the blood flow penumbra was larger than the structural penumbra, while in PVWMHs, the blood flow penumbra was almost the same as the structural penumbra.

## Introduction

Cerebral small vessel disease (CSVD) is the most common, chronic, and progressive vascular disease. It is the umbrella term used to describe pathologies of the vascular structures (small arteries, arterioles, capillaries, small veins, and venules) that are located in the brain parenchyma or the subarachnoid space [1, 2]. It is primarily responsible for stroke incidents, gait disturbances, depression, cognitive impairment, and dementia in the elderly [3]. White matter hyperintensities (WMHs) are high T2-weighted signal lesions that are one of the major imaging features of CSVD and are associated with the risk of stroke, dementia, or death [3]. A longitudinal study demonstrated that baseline WMHs were associated with the progression of CSVD and a decline in executive function [2].

Increasing evidence has shown that some tissue changes will also occur around WMHs. Although these changes cannot be detected by conventional magnetic resonance imaging (MRI) scanning, they will also cause some functional changes [4]. The specific, subtly changed normal-appearing white matter (NAWM) surrounding WMHs is called a “white matter hyperintensity penumbra (WMH-P)” in several studies [5–7]. The tissue changes in the NAWM may play an even more important clinical role in terms of clinical symptomatology [8]. The disruption of the microstructure in NAWM at baseline was shown to be associated with the future volume of the growing WMH. Some studies showed that arterial spin labeling (ASL) and diffusion tensor imaging (DTI) can reveal the tissue changes in the normal area of conventional MRI by assessing the blood supply and the structural damage severity of the penumbra, although the results are not completely consistent [5, 7, 9]. Another positron emission computed tomography (PET) study showed that the main changes occurring in NAWM are a decrease in metabolism, while its microstructure remains relatively intact [10]. One study showed that even in young patients with low disease burden, all measured structural, diffusion and physiological MR imaging parameters in the penumbra have statistically marked abnormalities [11]. Therefore, some scholars have proposed that WMHs constitute only “the tip of the iceberg,” are only a crude estimate of white matter injury and should not be dissociated from the somewhat more subtle white matter impairments in white matter integrity in the WMH-P [12]. Identifying and evaluating this specific and subtle change in the penumbra may affect early treatment decisions. It may be a novel treatment target that, if salvaged, might modify the time course of progressive WMH development. The axial diffusivity and the radial diffusivity can be obtained by DTI. DKI is a higher order diffusion model that is a straightforward extension of DTI [13] and is sensitive to the restricted and non-Gaussian diffusion effects in evaluating brain tissue microstructural complexity [14]. The DKI can provide measures such as the mean kurtosis, axial kurtosis, and radial kurtosis. At present, there is no study on the change in WMH-P structure using DKI.

The aim of this study was to examine WMH-P using DKI parameters and axial and radial parameters derived from DTI/DKI, which have not been examined before, to identify early microstructural changes. At the same time, we detected blood supply alterations in the penumbra using ASL, thereby facilitating our understanding of the mechanisms that result in WMHs.

## Materials and methods

### Subjects

CSVD subjects were recruited from patients who were admitted to the Neurology Department between August 2021 and January 2022. All patients underwent brain MRI due to clinically suspected cerebrovascular disease. The diagnosis of CSVD relied on imaging findings including white matter hyperintensities, lacunar ischemic stroke, lacunes, microbleeds, and visible perivascular spaces [15]. The inclusion criteria were as follows: (1) age ≥ 50 years; (2) cranial MRI confirming the presence of CSVD; and (3) patients with modified Fazekas stage 1 or 2 WMH [16]. Those findings were evaluated by two radiologists (Wang X. and Gao D.Y. with 11 and 3 years of experience in neuroradiology, respectively) in consensus. The exclusion criteria were as follows: (1) patients with specific causes of WMH (e.g., metabolic, toxic, infectious, multiple sclerosis, brain irradiation); (2) patients with a history of stroke; (3) patients with severe medical diseases, such as heart diseases, liver diseases, renal failure, tumors, or other systemic diseases; (4) patients with major depression or severe cognitive impairment (inability to perform the neuropsychological test or undergo the whole MRI scan); and (5) MRI safety contraindications and claustrophobia. Finally, 42 right-handed CSVD patients were included in this study. Subjects’ ages ranged from 50 to 80 years old (mean age was 66 years old; standard deviation was 7.23 years old). Table 1 describes the participant characteristics.

**Table 1:** Summary of participant characteristics

Variables	Mean (SD)
Number of subjects	42
Age (years)	66 (7.23)
Female (%)	57.14
Subjects with history of hypertension (%)	28.57
Subjects with history hypercholesterolemia (%)	21.43
Subjects with history diabetes (%)	14.29
Subjects with history smoking (%)	11.9

### Ethics statements

This study was approved by the medical ethics committee. Written informed consent was obtained from all participants or their legally acceptable representative.

### MRI acquisition

All MRI data were obtained using a 3.0T MRI scanner (Philips Ingenia CX R6.1, Philips Healthcare, Best, The Netherlands) equipped with a 32-channel phased array head coil. Each subject underwent three-dimensional (3D) T_1_ high-resolution imaging and T_2_-FLAIR scans. All subjects underwent DKI scans and three-dimensional pulsed continuous arterial spin labeling (3D-pCASL) scans. The parameters of each sequence were as follows: (1) sagittal 3D T_1_ high-resolution imaging [repetition time (TR) = 6.7 ms, echo time (TE) = 3.0 ms, inversion time (TI) = 596.7 ms, flip angle = 8◦, slice thickness = 1.0 mm, number of slices = 340, gap = 0, field of view (FOV) = 240 mm × 240 mm, and matrix = 240 × 240]; (2) axial 3D T_2_-FLAIR (TR = 4800 ms, TE = 309 ms, TI = 1650 ms, FOV = 240 mm × 240 mm, matrix = 240 × 240, slice thickness = 1 mm, and number of slices = 286; (3) 3D-pCASL perfusion images were acquired using 3D fast spin-echo acquisition with background suppression and with a post labeling delay of 2000 ms (TR = 4342 ms, TE = 10 ms, FOV = 240 mm × 240 mm, slice thickness = 6 mm, flip angle = 90°, the in-plane resolution = 3.75 mm × 3.75 mm, number of signals averaged (NSA) = 1, and number of slices = 18), the cerebral blood flow (CBF) images were automatically generated after scanning; and (4) DKI (TR = 3,880 ms, TE = 87 ms, slice thickness = 2 mm, gap = 0, FOV = 240 mm × 240 mm, number of slices = 60, matrix = 120 × 118, and 64 diffusion-weighted directions with b value = 0, 1000, and 2000 s/mm^2^).

### Image processing and analysis

#### Anatomical data preprocessing

The T_1_-weighted images were preprocessed using fsl_anat (https://fsl.fmrib.ox.ac.uk/fsl/fslwiki/fsl_anat), which provides a general pipeline for processing anatomical images. Most of the pipeline involves standardized use of FMRIB’s Software Library (FSL) tools [17, 18], including reorientation to the standard Montreal Neurosciences Institute (MNI) orientation, automatic cropping, bias-field correction, registration to standard space, brain extraction, and tissue-type segmentation.

#### Diffusion data preprocessing and DKI fitting

The preprocessing of each participant’s diffusion-weighted imaging (DWI) data was performed using the following steps: image denoising, removal of Gibbs ringing artifacts, motion and eddy current distortion correction accompanied by slice outlier replacement and slice-to-volume alignment, and bias-field correction. The voxel-wise calculation of the diffusion metrics was performed using Dipy [19–21], which yielded maps of the fractional anisotropy (FA), mean diffusivity (MD), axial diffusivity (AD), radial diffusivity (RD), mean kurtosis (MK), axial kurtosis (AK), and radial kurtosis (RK).

#### Lesion segmentation

WMHs were segmented by the lesion growth algorithm [22] as implemented in the lesion segmentation toolbox (LST) version 3.0.0 (www.statistical-modeling.de/lst.html) for spatial pyramid matching (SPM). Then, WMH clusters were separated into PVWMHs and DWMHs according to the “continuity to ventricle rule.” PVWMHs were defined as WMHs that were continuous with the margin of the lateral ventricle, and all others were defined as DWMHs after removing subcortical white matter. Finally, probabilistic maps for WMH and WM were processed with binarization.

#### Spatial relationship between WMH and NAWM

**Fig. 1:**
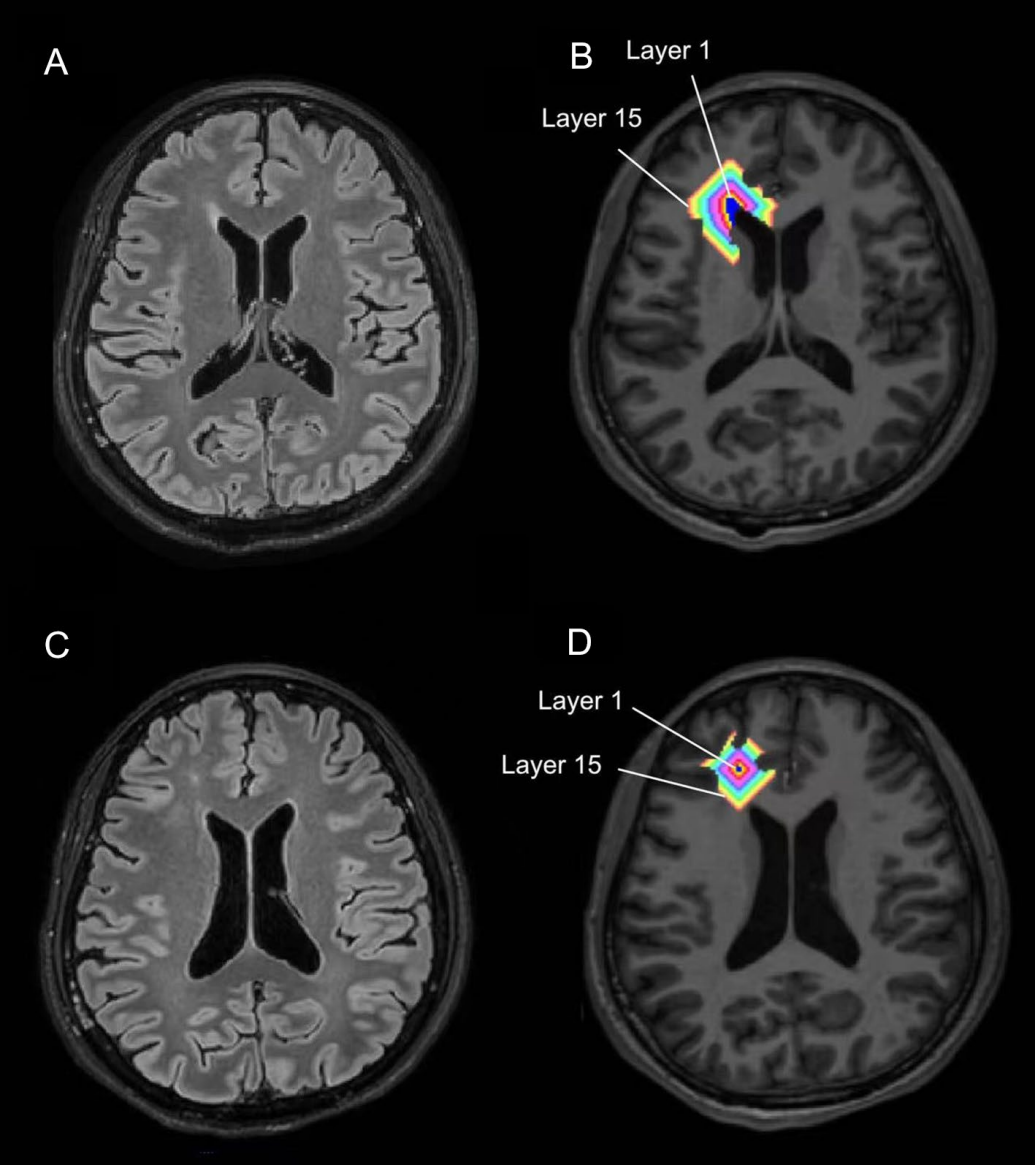
The spatial relationship between PVWMH, DWMH, and their corresponding NAWM layer masks. The dark blue represents WM lesions. The innermost yellow layer surrounding the WMH represents layer 1, and the outermost red layer represents layer 15. Each color represents a layer. **A** Periventricular white matter hyperintensity (PVWMH) on T_2_-FLAIR weight image (WI); **B** WMH and NAWM layer masks for PVWMH; **C** deep white matter hyperintensity (DWMH) on T_2_-FLAIR WI; **D** WMH and NAWM layer masks for DWMH

To assess the WMH penumbras for each imaging measure, an NAWM layer mask for each individual dataset was created by linearly aligning the defined binary WMHs to the T_1_-weighted images according to previous studies [5–7]. The NAWM layer mask consisted of 15 layers of PVWMH and DWMH separately. Each layer was parallel and gradually dilated away from the WMH by 1 mm. The innermost layer, closest to the WMH, was defined as layer 1 (NAWM-L1), and the outermost layer was layer 15 (NAWM-L15). To prevent overlapping between layers of neighboring WMHs, the WMH and previous NAWM layers were merged to create a new “WMH” before creating the next layer. To reduce the partial volume effects of the gray matter (GM) and cerebrospinal fluid (CSF), the GM and CSF maps were dilated by 2 voxels and subtracted from the NAWM layers (Fig. 1).

The CBF, FA, MD, AD, RD, MK, AK, and RK maps for each subject were coregistered to the corresponding individual 3D T_1_-weighted images.

**Fig. 2:**
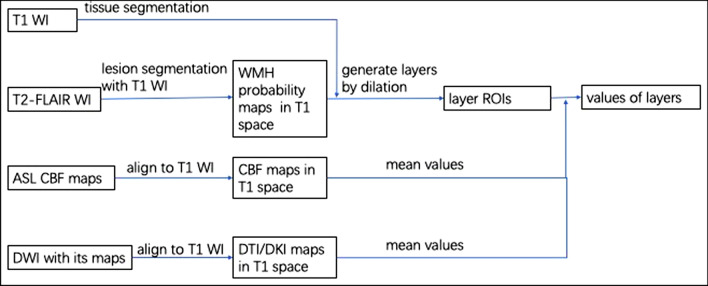
Schematic diagram showing the research methods. *WI* weight image; *ASL* arterial spin labeling; *CBF* cerebral blood flow; *DWI* diffusion-weighted imaging; *WMH* white matter hyperintensity; *DTI* diffusion tensor imaging; *DKI* diffusion kurtosis imaging; *ROI* region of interest

For each subject, the NAWM layer mask was applied to the ASL and DTI/DKI-related maps that were previously coregistered to individual 3D T_1_-weighted images. Later, the mean CBF, FA, MD, AD, RD, MK, AK, and RK values of each NAWM layer for the PVWMH and DWMH were computed. Similarly, the imaging parameter values of the PVWMH, DWMH and whole-brain NAWM were obtained (Fig. 2). The quality control of the WMH area and NAWM layer mask was completed by two radiologists (Wang X. and Gao D.Y. with 11 and 3 years of experience in neuroradiology, respectively) in consensus.

### Statistical analysis

The analyses were performed using IBM SPSS Statistics 23 and R version 4.2.1. To determine the extent of WMH CBF penumbras, the mean CBF value of the WMH and NAWM layers (L1–L15) was compared with the corresponding mean CBF value of the whole-brain NAWM using a paired *t* test. The first layer whose CBF value was not significantly different from that of the whole-brain NAWM was defined as the outer boundary of the CBF penumbra. There is a “location effect” of white matter association and commissural fibers surrounding the ventricles resulting in high DTI-FA in the NAWM surrounding the PVWMH compared with the mean NAWM DTI-FA of the entire brain [23]. Because of this anatomical phenomenon, the extent of the WMH structural penumbra was determined by comparing every two adjacent NAWM layers using paired *t* tests. The first of two neighboring layers whose values were not significantly different from each other was defined as the outer boundary of the structural penumbra. The PVWMH and DWMH were analyzed separately. A *p* value of ≤ 0.05 was set to indicate statistically significant differences.

## Results

**Fig. 3:**
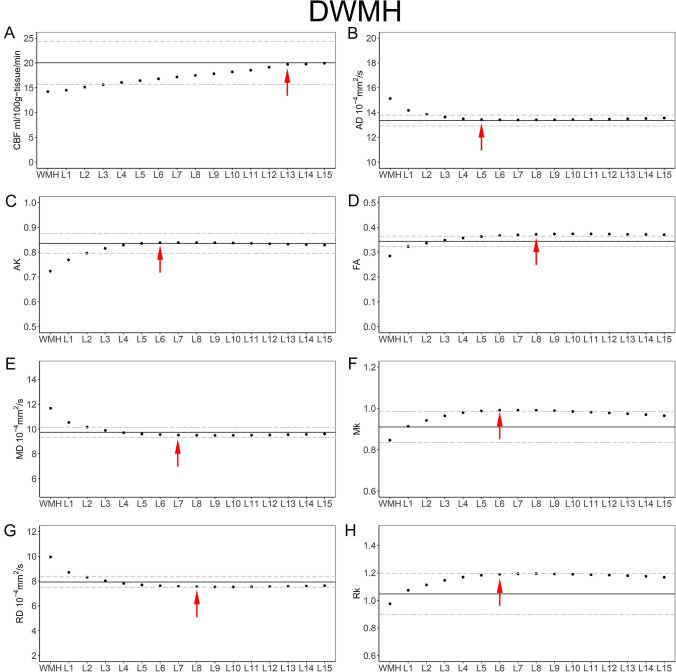
Group means of the DWMH and their outer NAWM layers. The solid horizontal and dotted lines represent the mean and standard error of the whole-brain NAWM CBF, AD, AK, FA, MD, MK, RD and RK values, respectively. Red arrows represent the outer boundary of the WMH penumbra for each dataset. **A** DWMH-CBF; **B** DWMH-AD; **C** DWMH-AK; **D** DWMH-FA; **E** DWMH-MD; **F** DWMH-MK; **G** DWMH-RD; **H** DWMH-RK

**Fig. 4:**
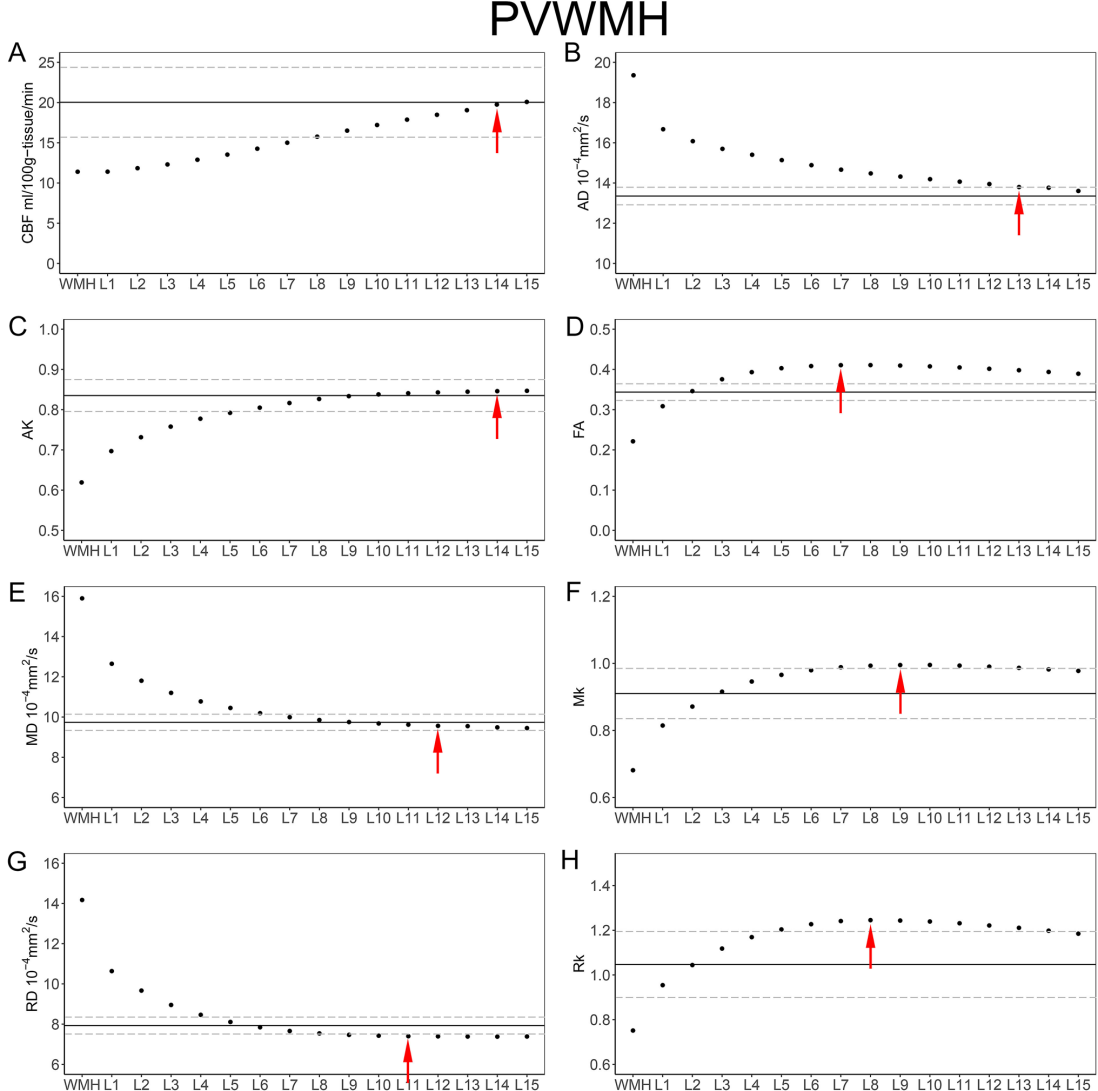
Group means of the PVWMH and their outer NAWM layers. The solid horizontal and dotted lines represent the mean and standard error of the whole-brain NAWM CBF, AD, AK, FA, MD, MK, RD and RK values, respectively. Red arrows represent the outer boundary of the WMH penumbra for each dataset. **A** PVWMH-CBF; **B** PVWMH-AD; **C** PVWMH-AK; **D** PVWMH-FA; **E** PVWMH-MD; **F** PVWMH-MK; **G** PVWMH-RD; **H** PVWMH-RK

The mean AD, AK, FA, MD, MK, RD, RK and CBF values of each NAWM layer of the DWMH are presented in Table 2 and Fig. 3. The mean AD, AK, FA, MD, MK, RD, RK and CBF values of each NAWM layer of the PVWMH are presented in Table 3 and Fig. 4.

**Table 2:** The mean CBF, AD, AK, FA, MD, MK, RD and RK values of the DWMH and their corresponding layers (mean ± SD)

	CBF (ml/100 g-tissue/min)	AD (10^−4^ mm^2^/s)	AK	FA	MD (10^−4^ mm^2^/s)	MK	RD (10^−4^ mm^2^/s)	RK
WMH	14.212 ± 5.708	15.130 ± 1.472	0.724 ± 0.079	0.285 ± 0.041	11.677 ± 1.229	0.846 ± 0.078	9.951 ± 1.192	0.976 ± 0.119
Layer 1	14.492 ± 5.219	14.175 ± 1.200	0.769 ± 0.063	0.323 ± 0.035	10.529 ± 0.891	0.913 ± 0.065	8.706 ± 0.820	1.074 ± 0.131
Layer 2	15.125 ± 4.893	13.8547 ± 1.081	0.796 ± 0.061	0.337 ± 0.032	10.157 ± 0.789	0.942 ± 0.069	8.312 ± 0.718	1.113 ± 0.146
Layer 3	15.626 ± 4.731	13.636 ± 1.039	0.815 ± 0.063	0.348 ± 0.031	9.894 ± 0.771	0.964 ± 0.075	8.023 ± 0.707	1.146 ± 0.161
Layer 4	16.066 ± 4.595	13.492 ± 1.008	0.829 ± 0.066	0.357 ± 0.029	9.705 ± 0.734	0.979 ± 0.079	7.812 ± 0.661	1.169 ± 0.173
Layer 5	16.439 ± 4.482	13.430 ± 0.999	0.835 ± 0.065	0.363 ± 0.028	9.605 ± 0.723	0.987 ± 0.082	7.693 ± 0.645	1.183 ± 0.180
Layer 6	16.799 ± 4.361	13.410 ± 0.997	0.838 ± 0.062	0.367 ± 0.028	9.553 ± 0.713	0.991 ± 0.085	7.624 ± 0.631	1.190 ± 0.185
Layer 7	17.166 ± 4.254	13.399 ± 0.971	0.839 ± 0.059	0.370 ± 0.028	9.517 ± 0.686	0.991 ± 0.086	7.575 ± 0.607	1.194 ± 0.187
Layer 8	17.489 ± 4.182	13.408 ± 0.950	0.839 ± 0.058	0.372 ± 0.028	9.500 ± 0.658	0.990 ± 0.087	7.545 ± 0.581	1.194 ± 0.188
Layer 9	17.833 ± 4.131	13.413 ± 0.926	0.838 ± 0.056	0.373 ± 0.029	9.489 ± 0.635	0.988 ± 0.086	7.527 ± 0.565	1.192 ± 0.188
Layer 10	18.197 ± 4.100	13.426 ± 0.925	0.837 ± 0.055	0.374 ± 0.030	9.493 ± 0.643	0.985 ± 0.086	7.527 ± 0.582	1.190 ± 0.199
Layer 11	18.542 ± 4.116	13.446 ± 0.937	0.836 ± 0.053	0.374 ± 0.030	9.506 ± 0.654	0.982 ± 0.087	7.536 ± 0.595	1.187 ± 0.199
Layer 12	19.145 ± 4.174	13.471 ± 0.948	0.834 ± 0.052	0.373 ± 0.031	9.527 ± 0.677	0.978 ± 0.088	7.555 ± 0.623	1.184 ± 0.199
Layer 13	19.719 ± 4.167	13.492 ± 0.946	0.832 ± 0.051	0.372 ± 0.031	9.549 ± 0.690	0.974 ± 0.087	7.577 ± 0.643	1.179 ± 0.187
Layer 14	19.762 ± 4.293	13.523 ± 0.972	0.831 ± 0.049	0.372 ± 0.032	9.574 ± 0.706	0.970 ± 0.087	7.600 ± 0.658	1.175 ± 0.185
Layer 15	19.943 ± 4.388	13.562 ± 1.018	0.829 ± 0.049	0.371 ± 0.033	9.611 ± 0.752	0.964 ± 0.086	7.636 ± 0.701	1.168 ± 0.183
NAWM	20.0374.343	13.353 ± 0.437	0.835 ± 0.040	0.344 ± 0.021	9.737 ± 0.403	0.910 ± 0.075	7.929 ± 0.421	1.047 ± 0.148

*CBF* cerebral blood flow, *AD* axial diffusion, *AK* axial kurtosis, *FA* fractional anisotropy, *MD* mean diffusivity, *MK* mean kurtosis, *RD* radial diffusion, *RK* radial kurtosis, *WMH* white matter hyperintensity, *NAWM* normal-appearing white matter, *SD* standard deviation

**Table 3:** The mean CBF, AD, AK, FA, MD, MK, RD and RK values of the PVWMH and their corresponding layers (mean ± SD)

	CBF(ml/100 g-tissue/min)	AD (10^−4^ mm^2^/s)	AK	FA	MD (10^−4^ mm^2^/s)	MK	RD (10^−4^ mm^2^/s)	RK
WMH	11.399 ± 3.784	19.356 ± 1.593	0.619 ± 0.057	0.221 ± 0.031	15.900 ± 1.371	0.681 ± 0.052	14.172 ± 1.336	0.751 ± 0.084
Layer 1	11.406 ± 3.414	16.673 ± 0.906	0.697 ± 0.048	0.309 ± 0.038	12.648 ± 0.700	0.815 ± 0.064	10.635 ± 0.768	0.954 ± 0.131
Layer 2	11.840 ± 3.246	16.080 ± 0.690	0.731 ± 0.045	0.346 ± 0.036	11.804 ± 0.620	0.871 ± 0.069	9.667 ± 0.728	1.044 ± 0.146
Layer 3	12.306 ± 3.239	15.698 ± 0.586	0.758 ± 0.046	0.376 ± 0.031	11.201 ± 0.553	0.916 ± 0.070	8.953 ± 0.645	1.118 ± 0.157
Layer 4	12.891 ± 3.299	15.404 ± 0.555	0.777 ± 0.048	0.393 ± 0.028	10.776 ± 0.506	0.946 ± 0.068	8.462 ± 0.574	1.169 ± 0.161
Layer 5	13.531 ± 3.381	15.138 ± 0.569	0.792 ± 0.049	0.403 ± 0.028	10.451 ± 0.482	0.966 ± 0.068	8.107 ± 0.530	1.204 ± 0.164
Layer 6	14.263 ± 3.502	14.888 ± 0.573	0.805 ± 0.049	0.408 ± 0.029	10.191 ± 0.475	0.980 ± 0.070	7.843 ± 0.524	1.227 ± 0.169
Layer 7	15.009 ± 3.634	14.663 ± 0.574	0.817 ± 0.048	0.411 ± 0.031	9.992 ± 0.474	0.989 ± 0.071	7.657 ± 0.527	1.241 ± 0.174
Layer 8	15.760 ± 3.788	14.477 ± 0.565	0.827 ± 0.046	0.411 ± 0.032	9.852 ± 0.459	0.993 ± 0.072	7.539 ± 0.521	1.245 ± 0.177
Layer 9	16.509 ± 3.955	14.319 ± 0.545	0.834 ± 0.046	0.410 ± 0.033	9.750 ± 0.438	0.995 ± 0.072	7.465 ± 0.508	1.243 ± 0.178
Layer 10	17.209 ± 4.128	14.191 ± 0.519	0.838 ± 0.046	0.408 ± 0.034	9.679 ± 0.406	0.995 ± 0.072	7.423 ± 0.485	1.239 ± 0.180
Layer 11	17.875 ± 4.302	14.066 ± 0.507	0.841 ± 0.046	0.405 ± 0.034	9.621 ± 0.392	0.993 ± 0.073	7.398 ± 0.477	1.231 ± 0.182
Layer 12	18.481 ± 4.449	13.949 ± 0.513	0.843 ± 0.046	0.402 ± 0.035	9.562 ± 0.384	0.990 ± 0.075	7.390 ± 0.477	1.221 ± 0.184
Layer 13	19.050 ± 4.613	13.799 ± 0.539	0.845 ± 0.046	0.398 ± 0.035	9.548 ± 0.382	0.987 ± 0.076	7.382 ± 0.474	1.211 ± 0.183
Layer 14	19.753 ± 4.708	13.765 ± 0.523	0.846 ± 0.046	0.394 ± 0.036	9.485 ± 0.393	0.982 ± 0.077	7.377 ± 0.478	1.198 ± 0.180
Layer 15	20.079 ± 4771	13.605 ± 0.551	0.847 ± 0.045	0.389 ± 0.037	9.450 ± 0.403	0.978 ± 0.078	7.383 ± 0.483	1.185 ± 0.178
NAWM	20.0374.343	13.353 ± 0.437	0.835 ± 0.040	0.344 ± 0.021	9.737 ± 0.403	0.910 ± 0.075	7.929 ± 0.421	1.047 ± 0.148

*CBF* cerebral blood flow, *AD* axial diffusion, *AK* axial kurtosis, *FA* fractional anisotropy, *MD* mean diffusivity, *MK* mean kurtosis, *RD* radial diffusion, *RK* radial kurtosis, *WMH* white matter hyperintensity, *NAWM* normal-appearing white matter, *SD* standard deviation

The extents of the DWMH penumbras were as follows: 5 mm for AD penumbras; 6 mm for AK penumbras; 8 mm for FA penumbras; 7 mm for MD penumbras; 6 mm for MK penumbras; 8 mm for RD penumbras; 6 mm for RK penumbras; and 13 mm for the CBF penumbras (Fig. 3). The maximum range of the structural penumbra was 8 mm.

The extents of the PVWMH penumbras were as follows: 13 mm for AD penumbras; 14 mm for AK penumbras; 7 mm for FA penumbras; 12 mm for MD penumbras; 9 mm for MK penumbras; 11 mm for RD penumbras; 8 mm for RK penumbras; and 14 mm for the CBF penumbra (Fig. 4). The maximum range of the structural penumbra was 14 mm.

## Discussion

Our finding is that the range of the structural penumbra of DWMHs is 8 mm and that of PVWMHs is 14 mm. Previous DTI studies have shown that the structural penumbra of WMHs ranges from 2 to 9 mm [5, 7]. The AD describes the mean diffusion coefficient of water molecules diffusing parallel to the tract within the voxel of interest [24]. The RD can be defined as the magnitude of water diffusion perpendicular to the tract [24]. Axial and radial diffusivity could be DTI markers of axonal and myelin damage, respectively [24]. The mean kurtosis (MK) is the directionally averaged kurtosis and has been shown to be useful in assessing pathophysiological changes, thus yielding another dimension of information to characterize water diffusion in biological tissues [25]. Axial and radial kurtoses (AK and RK) measured the kurtoses along the directions parallel and perpendicular, respectively, to the principal diffusion direction [25]. Regarding the penumbras of PVWMH, our results show that the structural penumbra ranges are 13 mm for AD, 11 mm for RD, and 14 mm for AK. These parameters had not been considered in the past, and we showed larger penumbras in this study than were shown in previous studies [5, 7]. The maximum penumbra range was displayed by AK. This may be because AK is obtained from DKI based on a non-Gaussian model, which can more accurately approximate diffusion-weighted signal attenuation [13]. In this study, the ranges of PVWMH and DWMH displayed in MD were 12 and 7 mm, respectively. In previous studies the MD ranges for PVWMH were 6 and 5 mm, and the MD ranges for DWMH were 2 and 5 mm [5, 7]. The MD in this study showed a wider range of structural penumbra than was previously reported. This may be because the DKI scan is different from the previous DTI scan parameters. DTI/DKI parameters have been found to be very sensitive in identifying some alterations that characterize many neurological diseases [26, 27], and DKI is even more suited to characterize these early subtle alterations in CSVD [28]. The kurtosis index appeared to provide increased sensitivity to group differences that occur in areas with complex fiber arrangements [13]. CSVD involves a series of complex physiological and pathological changes, and its pathological tissue changes vary in type and severity. In early stages, tissue alterations can be subtle, especially when they occur away from visible lesions [28]. Pathologically, NAWM may correspond to mild tissue changes with a slightly lower myelin density, an activated endothelium, a looser but still largely intact axonal network and a normal glial density [29]. Different parameters have different structural anomaly detection capabilities. Further work relating these imaging biomarkers to histopathologic findings, especially at early stages in CSVD, is required to fully understand the pathological processes responsible for white matter damage within and around WMHs.

The results of this study show that the extent of the CBF penumbras of DWMH is 13 mm, and the extent of the structural penumbra is 8 mm. Previous evidence demonstrated that the CBF WMH penumbra extends approximately 7–14 mm distal to WMH lesions, whereas the structural WMH penumbra, as measured by DTI, extends from 2 to 9 mm, suggesting that decreased CBF may precede microstructural deterioration of NAWM tissue [5, 7]. In DWMH-P, we also found that the penumbra of CBF was larger than the penumbra of structure. Pathologically, it has been shown that the density of small afferent vessels is not only decreased in WMH but extends into NAWM and the cortex [30]. From hemodynamic-based measures, some studies provide evidence for global hemodynamic effects in relation to CSVD [31, 32], and the decrease in CBF in nature may be partly responsible for the impairment of white matter. WMH progression is likely due to demyelinating injury secondary to low perfusion [33]. Our study also suggests that decreased blood flow precedes structural changes in DWMH-P if subtle structural changes are assessed by DTI/DKI.

The results of this study show that both the blood flow and structural penumbras in PVWMHs have extents of 14 mm. Unlike the blood flow penumbra of DWMH, which is larger than the structural penumbra, the blood flow penumbra of PVWMH is almost the same as the structural penumbra. First, this may be due to the original structural or hemodynamic differences in the normal periventricular white matter and deep white matter originally, resulting in differences in WMH penumbras [34, 35]. A recent study found that the FA values are lower in PVWMHs than in DWMHs, suggesting the presence of different mechanistic contributions to WMHs based on location [36]. Second, this may also be due to different etiologies leading to different pathophysiology. The etiopathogenesis of CSVD reflects several mechanisms. It mainly includes blood‒brain barrier dysfunction, impaired vasodilation, vessel stiffening, dysfunctional blood flow and interstitial fluid drainage, white matter rarefaction, ischemia, inflammation, myelin damage, and secondary neurodegeneration [37]. We speculate that a variety of factors lead to structural damage in PVWMH-P, not just reduced perfusion. This is consistent with previous pathologists' hypothesis that DWMH changes present more hypoxic/ischemic damage, whereas PVWMH changes may have a greater inflammatory/metabolic component [38, 39]. Finally, it may also be related to the histopathological differences between the two sites. PVWMHs are characterized by gliosis, loosening of the white matter fibers, and myelin loss, while DWMHs may present less gliosis but more axonal loss, vacuolization, and arteriolosclerosis [40].

WMH progression is traditionally thought to be continuous and uniform; it is now known to be a dynamic and highly variable process that sometimes regresses [41, 42]. Although we have not conducted longitudinal studies, it is worth mentioning that some longitudinal studies have found that the changes in penumbra microstructure are related to progress, which means that there are new targets for treatment [10, 43]. The changes in the penumbra signal may further our understanding of the underlying etiology of early WMH development and expansion. This is crucial to preventing WMH growth and the subsequent development of cognitive and motor impairment. Finding early reversible alterations would help to identify a biomarker for trials designed with the objective of slowing or reversing the effects of WMH development.

## Limitations

There are several limitations in this study. First, the sample size of this study was relatively small, and it was a cross-sectional study. In the future, we should increase the sample size and conduct a longitudinal study. Second, even though the blood flow and structure of the white matter may differ from region to region, this study only measured the average NAWM layer equidistant from a WMH. Third, part of the NAWM layer extended into the subcortical white matter while the microstructure could be different between deep and subcortical white matter [44]. This is also a limitation of this study. Fourth, the CBF and DTI/DKI maps with lower spatial resolution were registered to 3D T_1_WI with higher spatial resolution, so the values of the CBF and DTI/DKI parameters might not be the true values associated with each voxel. Finally, it is also necessary to further study the change in WMH-P signaling and its relationship with clinical manifestations such as cognitive status and motor function.

## Conclusion

In conclusion, DTI/DKI and ASL can show the structural and blood flow changes in brain tissue surrounding WMHs, indicating that the scope of WM injury extends beyond visible lesions commonly observed on conventional MRI. In DWMH-P, the CBF penumbra is larger than the structural penumbra, but in PVWMH-P, the ranges of the CBF and structural penumbra extents are almost the same.
